# Hypoxia-preconditioned olfactory mucosa mesenchymal stem cells abolish cerebral ischemia/reperfusion-induced pyroptosis and apoptotic death of microglial cells by activating HIF-1α

**DOI:** 10.18632/aging.103307

**Published:** 2020-06-07

**Authors:** Yan Huang, Fengbo Tan, Yi Zhuo, Jianyang Liu, Jialin He, Da Duan, Ming Lu, Zhiping Hu

**Affiliations:** 1 Key Laboratory of Protein Chemistry and Developmental Biology of Ministry of Education, College of Life Sciences, Hunan Normal University, Changsha 410081, Hunan, P.R. China; 2 Department of Neurosurgery, Second Affiliated Hospital of Hunan Normal University, Changsha 410003, Hunan, P.R. China; 3 Hunan Provincial Key Laboratory of Neurorestoration, Second Affiliated Hospital of Hunan Normal University, Changsha 410003, Hunan, P.R. China; 4 Department of Gastrointestinal Surgery, Xiangya Hospital, Central South University, Changsha 410008, Hunan, P.R. China; 5 Department of Neurology, The Second Xiangya Hospital, Central South University, Changsha 410011, Hunan, P.R. China

**Keywords:** hypoxia-preconditioned OM-MSCs, HIF-1α, microglial, pyroptosis, apoptosis

## Abstract

Microglial cells are the first line immune cells that initiate inflammatory responses following cerebral ischemia/reperfusion(I/R) injury. Microglial cells are also associated with a novel subtype of pro-inflammatory programmed cell death known as pyroptosis. Research has been directed at developing treatments that modulate inflammatory responses and protect against cell death caused by cerebral I/R. Key among such treatments include mesenchymal stem cell (MSC) therapy. A unique type of MSC termed olfactory mucosa mesenchymal stem cell (OM-MSC) confers neuroprotection by promoting the secretion of paracrine factors, and neuroprotection. This study investigated whether hypoxic OM-MSCs could inhibit microglial cell death upon I/R insult in *vitro*. A traditional oxygen-glucose deprivation/reperfusion (OGD/R) model, analogous to I/R, was established. Results showed that OGD/R induced apoptosis and pyroptosis in microglial cells while hypoxia in OM-MSCs significantly attenuated these effects. Moreover, the effects of OM-MSCs were mediated by Hypoxia-inducible factor 1-alpha (HIF-1α). Taken together, these findings reveal that hypoxia-preconditioned OM-MSC inhibits pyroptotic and apoptotic death of microglial cell in response to cerebral ischemia/reperfusion insult by activating HIF-1α in *vitro*.

## INTRODUCTION

Among central nervous system (CNS) diseases, cerebral ischemic stroke is the most common cause of death and disability [1]. This condition has been successfully treated with tissue plasminogen activator (tPA) therapy. However, the reperfusion process may result in serious brain tissue damage [2], and has been an intractable challenge in stroke treatment. It is, therefore, crucial to elucidate the latent mechanisms of cerebral I/R injury.

Cerebral I/R insult is often accompanied with inflammation [3]. In addition, cerebral I/R injury causes different subtypes of programmed cell death, including apoptosis and pyroptosis [4]. Pyroptosis is a new type of programmed cell death associated with inflammatory response [5]. Numerous studies have demonstrated that pyroptosis is extensively involved in CNS disorders [6–8]. It shares similar features with apoptotic cell death such as DNA fragmentation. On the other hand, pyroptosis is distinct from apoptosis, in that it is not regulated by the classical apoptotic-associated protein, caspase-3, but by inflammation-associated protein caspase-1 [7]. Studies have reported that cleaved-caspase1 regulates both apoptotic and pyroptotic cell death under oxidative stress condition [9]. Meanwhile, the inflammasome of NLRP3 plays a critical role in pyroptosis. Generally, cerebral I/R causes activation of NLPR3 inflammasome and secretion of proinflammation cytokines, including Interleukin-1β(IL-1β) and Interleukin-18(IL-18) which induces neuronal cells death [10]. However, information regarding microglia and their contribution to pyroptosis during cerebral I/R is still insufficient.

Microglia are resident macrophage cells that act rapidly to inhibit inflammation and scavenge for damaged cells or tissues [11, 12]. However, over-activation of microglia has been shown to induce production of pro-inflammatory mediators thereby aggravating inflammation responses [13, 14]. Furthermore, activation of microglia regulates the expression of pattern recognition receptor (PRR) proteins, such as NOD-like receptor (NLR) family, and pyrin domain-containing protein 3 (NLRP3), which is one of the most important inflammasome sensor genes related to pyroptosis [6, 15]. Targeting both pyroptotic and apoptotic cell death in microglia during cerebral I/R insult may, therefore, be a promising therapeutic approach.

Cell-based therapies such as MSCs have been suggested to be a meaningful strategy for therapy against inflammatory-associated diseases. This is because MSCs provide immune modulation and reparative property [16, 17]. MSCs can replace or restore damaged cells by releasing paracrine factors [18]. For instance, a source of MSCs derived from the human olfactory mucosa (OM) [19, 20]. In addition, we previously demonstrated that hypoxia pre-conditioning of OM-MSCs could regulating production of paracrine mediators by OM-MSCs, which conferred neuroprotection against cerebral I/R injury [21]. Other recent scientific evidence suggests that hypoxia preconditioning of MSCs is a beneficial approach to promote cell survival and resolve several diseases such as spinal cord injury (SCI) and cerebral ischemia [22]. Furthermore, HIF-1α, a protein was sensitive to oxidative concentration, has been reported to regulate responses to hypoxia [23]. However, to date, it is not known whether co-culturing OM-MSCs with microglia has a protective effect against either pyroptotic or apoptotic cell death during cerebral I/R injury.

In this study, we hypothesized that hypoxic preconditioning of OM-MSCs may prevent both pyroptosis and apoptosis of microglia during cerebral I/R injury in *vitro*. We established the traditional OGD/R model and simulated cerebral ischemia/reperfusion injury in BV2 microglial cells. The findings of our study are expected to provide meaningful insights into the therapeutic potential of cell-based approaches for ischemic stroke.

## RESULTS

### OGD/R induces apoptosis of BV2 microglial cells

**Figure 1:**
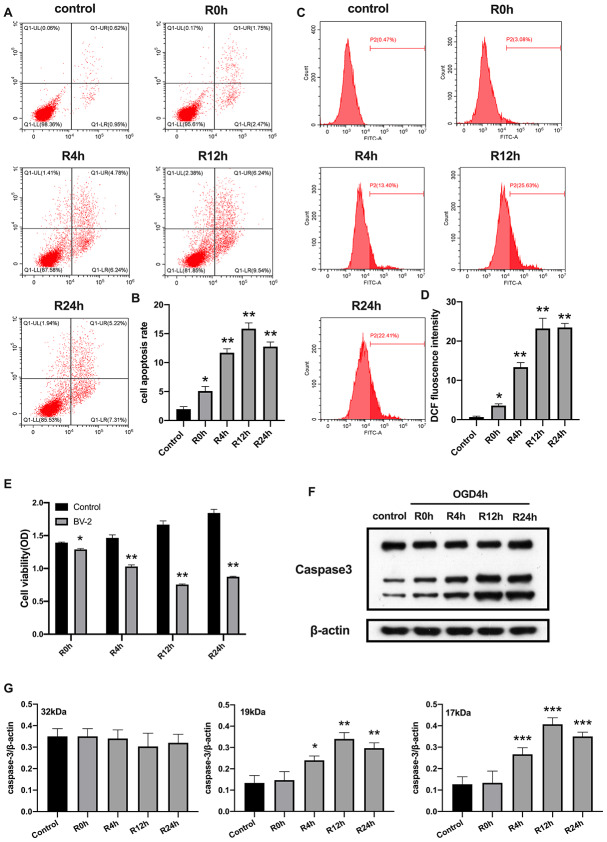
**OGD/R induced apoptosis of BV2 microglial cells.** (**A**, **B**) Flow cytometry with Annexin V/PI staining was used to assess cell apoptosis in BV2 microglial cells. (**C**, **D**) Flow cytometry was performed to measure the level of ROS in BV2 microglial cells. (**E**) The viability of BV2 microglial cells was determined by MTT assay. (**F**, **G**) The expression of caspase3 was quantified by western-blot analysis. All data are presented as the mean value ±SD. *p<0.05; **p<0.01, ***p<0.001, compared with control.

We first investigated whether OGD/R could induce apoptosis in BV2 microglial cells. As expected, in Figure 1, shows that the rate of cell apoptosis significantly increased after OGD/R injury, while cell viability was markedly reduced relative to the control group (Figure 1A, 1B and 1E). Moreover, rate of cell apoptosis and cell viability were highest and lowest at the 12^th^ hour of reperfusion, respectively. Studies indicate that ROS increases expression of damage-associated molecular patterns (DAMPs), thereby aggravating cell death during OGD/R insult [24]. We therefore measured the levels of ROS. Results showed that ROS level was significantly higher in cells subjected to OGD/R insult than in control group (Figure 1C, 1D). The expression of apoptosis marker, caspase-3, in BV2 microglial cells was measured using western-blot analysis. The result showed that caspase-3 expression significantly increased from 4-h, reaching the peak at 12-h, and then slowly dropped after 24-h of reperfusion (Figure 1F, 1G). Taken together, these results indicated that OGD/R induced apoptotic cell death in BV2 microglial cells.

### OGD/R causes NLRP3 inflammasome activation and pyroptosis in BV2 microglial cells

**Figure 2:**
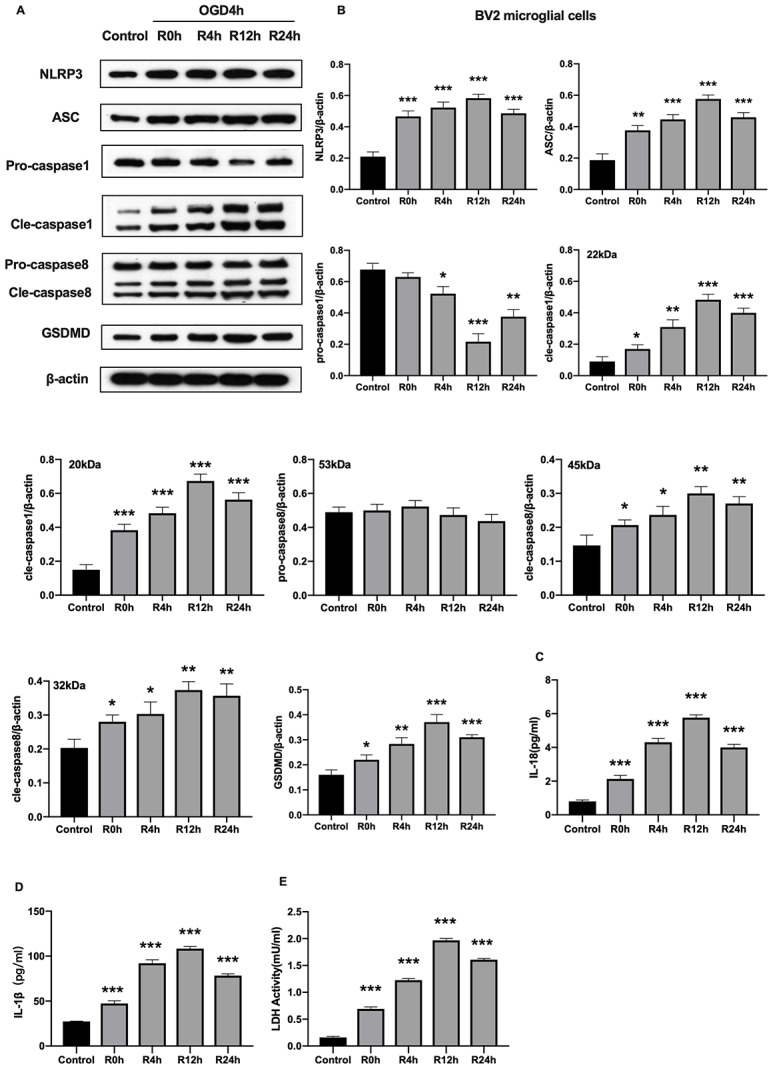
**OGD/R induced NLRP3 inflammasome activation and pyroptosis in BV2 microglial cells.** (**A**, **B**) Expression of NLRP3, ASC, pro-caspase1, cleaved-caspase1, pro-caspase8, cleaved-caspase8 and GSDMD in BV2 microglial cells as measured by western-blot analysis. (**C**, **D**) Level of IL-1β and IL-18 in BV2 microglial cells as determined by ELISA. (**E**) The level of LDH activity in BV2 microglial cells as measured by ELISA. All data are presented as the mean value ±SD. *p<0.05; **p<0.01, ***p<0.001, compared with control.

Unlike cerebral ischemia/reperfusion-induced apoptosis which has been studied extensively characterized in neurons and microglial cells, inflammation-associated pyroptotic cell death in BV2 microglial cells following cerebral ischemia/reperfusion injury has received little attention. Herein, we measured expression of inflammasome and pyroptosis-associated proteins in BV2 microglial cells under OGD/R condition. Results showed that NLRP3, ASC, GSDMD, pro-caspase1, cleaved-caspase1, and cleaved-caspase8 proteins were significantly higher in cells subjected to OGD/R insult relative to control group cells. In contrast, expression of pro-caspase8 was not altered after OGD injury for 6 hours or during reperfusion. The protein levels of all tested proteins, except pro-caspase1 and pro-caspase8, reached the peak at 12-h of reperfusion time point (Figure 2A, 2B). Given that activation of inflammasome and pyroptosis triggers the release of pro-inflammatory factors (IL-1β and IL-18), we next investigated whether these factors were altered after OGD/R. Figure 2C, 2D reveals that the level of IL-1β and IL-18 markedly increased in cells subjected to OGD/R relative to the control group cells. Pyroptosis is characterized by cellular swelling and rupture of the plasma membrane [7]. Thus, the loss of integrity of cellular plasma membrane and LDH activity were tested at various reperfusion time points following OGD/R insult. We noted a marked increase in the level of LDH activity at each reperfusion time points compared to the control group (Figure 2E). Furthermore, the peak level of all pro-inflammatory factors occurred at the 12^th^ hours of reperfusion after OGD insult. Collectively, these findings indicated that activation of NLRP3 inflammasome and pyroptosis occurred in BV2 microglial cells subjected to OGD/R.

### Co-culture of hypoxia-preconditioned OM-MSCs with BV2 microglial cells prevents apoptotic cell death in OGD/R insult context

MSCs secrete anti-inflammation cytokines and growth factors through secretion and paracrine mechanisms, which may influence the course of cerebral ischemic processes [25]. In this study, MSCs isolated from adult human olfactory mucosa (OM-MSCs) exhibited a typical fibroblastic cell pattern and were positively expressed in Nestin and STRO-1 by the immunofluorescence (Supplementary Figure 1A, 1B). Notably, these cells tested positive for CD44, CD73, CD90, CD105, CD133, and CD146, but were negative for CD34 and CD45 negative cells (Supplementary Figure 1C).

**Figure 3:**
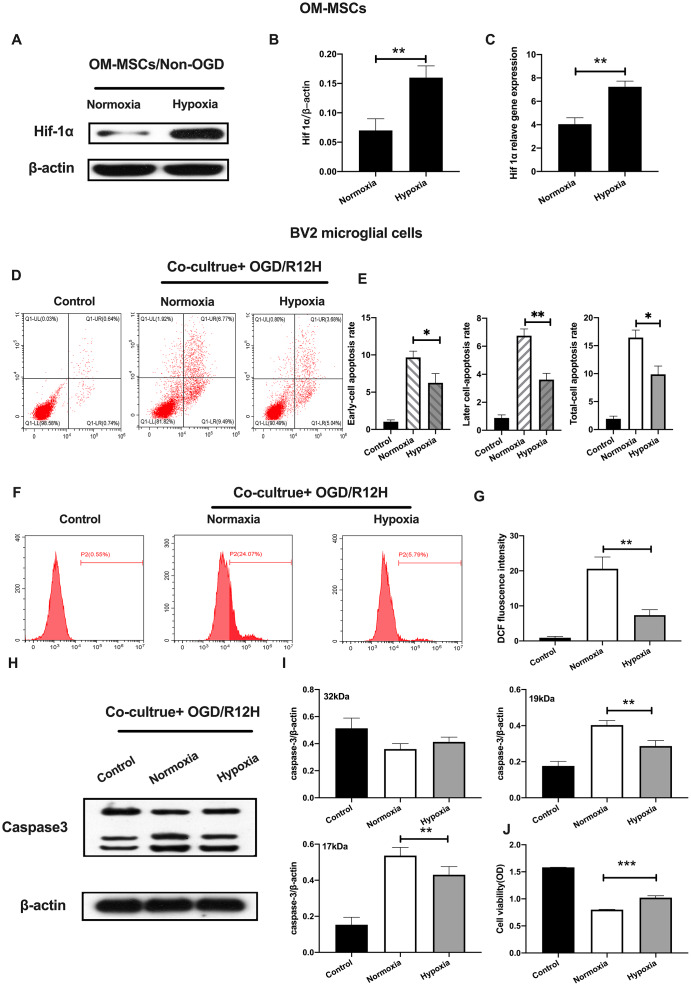
**Hypoxia-preconditioned OM-MSCs prevented cerebral OGD/R-induced apoptosis in BV2 microglial cells.** (**A**–**C**) Protein and mRNA expression of HIF-1α in OM-MSCs as determined by western-blot and qPCR, respectively. (**D**–**E**) The apoptosis rate of BV2 microglial cells as evaluated by flow cytometry with Annexin V/PI staining. (**F**, **G**) ROS generation in BV2 microglial cells as measured by flow cytometry. (**H**, **I**) The expression of caspase3 in BV2 microglial cells as determined by Western blotting analysis. (**J**) The viability of BV2 microglial cells as determined by MTT assay. All data are presented as the mean value ±SD. *p<0.05; **p<0.01, ***p<0.001, compared with normoxia group.

Previously, it was reported that hypoxia preconditioning promotes survival of transplanted MSCs. HIF-1α, plays an important role in hypoxia responses [26]. We, therefore, profiled the expression of HIF-1α expression under hypoxia conditions in OM-MSCs. Results showed the HIF-1α was significantly elevated in hypoxia pretreated OM-MSCs relative to normoxia treated OM-MSCs group (Figure 3A–3C). Next, we investigated whether hypoxia-preconditioned OM-MSCs could alleviate apoptotic cell death in BV2 microglial cells subjected to OGD/R. Having found that the effects of reperfusion were significant at 12-h, we chose OGD 4-h plus reperfusion for 12-h in the subsequent experiments. Figure 3 shows that hypoxia-preconditioned OM-MSCs significantly attenuated the rate of apoptosis in BV2 microglial cells subjected to OGD for 4-h followed by reperfusion for 12-h. In contrast, hypoxia-preconditioned OM-MSCs improved cell viability (Figure 3D–3E and 3J). Meanwhile, BV2 microglial cells co-cultured with hypoxia preconditioned OM-MSCs showed decreased ROS levels compared to the control group cells exposed to OGD/R for 12-h (Figure 3F, 3G). In addition, we noted a reduction in levels of caspase-3 in BV2 microglial cells co-cultured with hypoxia-preconditioned OM-MSCs relative to normoxia pretreated cells (Figure 3H–3I). Taken together, these findings confirmed that co-culture of BV2 microglial cells with hypoxia-preconditioned of OM-MSCs significantly potentiated anti-apoptotic cell death induced by OGD/R insult.

### Co-cultured of BV2 microglial cells with hypoxia-preconditioned OM-MSCs attenuates pyroptotic cell death induced by OGD/R insult

**Figure 4:**
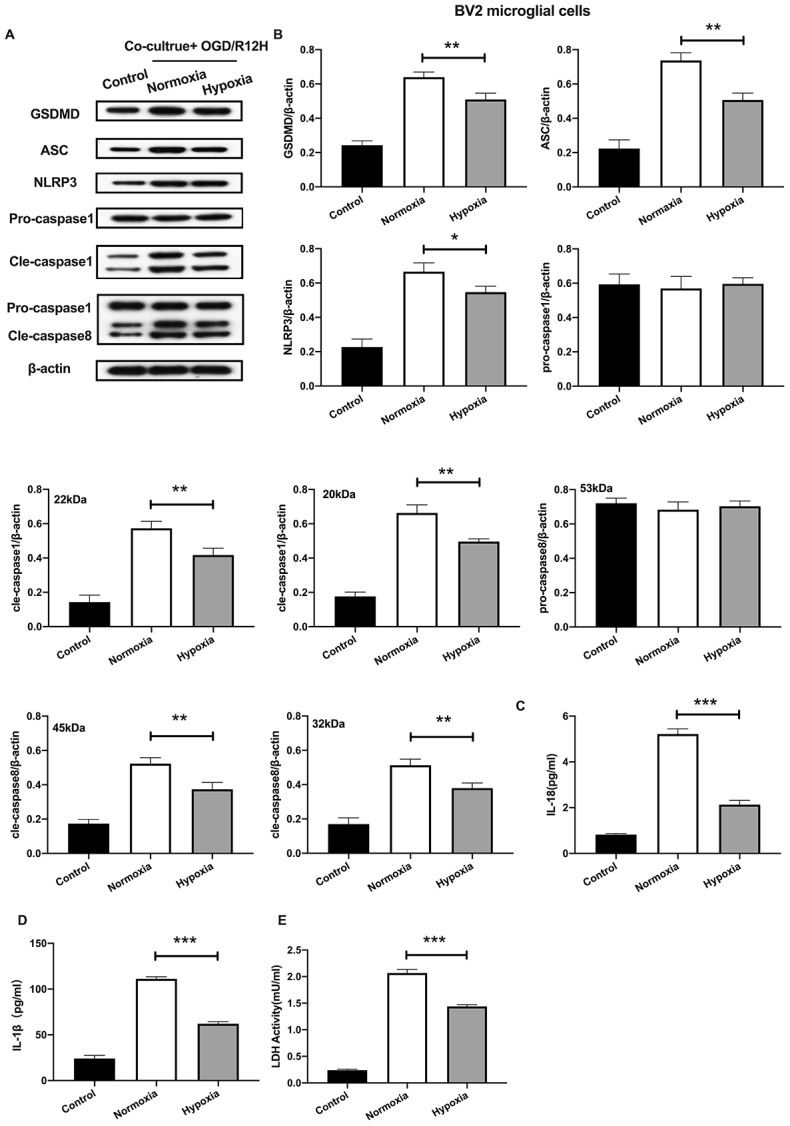
**Hypoxia-preconditioned OM-MSCs attenuated cerebral OGD/R-induced pyroptosis in BV2 microglial cells.** (**A**, **B**) Expression of NLRP3, ASC, pro-caspase1, cleaved-caspase1, pro-caspase8, cleaved-caspase8 and GSDMD in BV2 microglial cells as quantified by Western blotting analysis. (**C**, **D**) Level of IL-1β and IL-18 in BV2 microglial cells as measured using ELISA. (**E**) The level of LDH activity in BV2 microglial cells as measured by ELISA. All data are presented as the mean value ±SD. *p<0.05; **p<0.01, ***p<0.001, compared with normoxia group.

We further explored the functional role of hypoxia-preconditioned OM-MSCs on pyroptosis in BV2 microglial cells subjected to OGD/R injury. Pyroptotic cell death was evaluated by Western blotting and ELISA assay. Results showed that NLRP3 inflammasome and pyroptosis-associated proteins were lower in BV2 microglial cells co-cultured with hypoxia-preconditioned OM-MSCs cells relative to those co-cultured with normoxia pretreated OM-MSCs cells after treatment with OGD/R for 12-h. However, pro-caspase1 and pro-caspase8 were not significantly different between the two groups (Figure 4A, 4B). Of note, OGD/R for 12-h caused a marked reduction of IL-1β and IL-18 levels (Figure 4C, 4D). In addition, LDH activity was decreased in BV2 microglial cells exposed to OGD/R for 12-h co-cultured with hypoxia-preconditioned OM-MSCs relative to OM-MSCs preconditioned to normoxia (Figure 4E). Overall, these findings reveal that hypoxia-preconditioned OM-MSCs attenuated apoptosis or pyroptosis of BV2 microglial cells under OGD/R-induced injury.

### HIF-1α regulates apoptosis of BV2 microglial cells exposed to OGD/R conditions

**Figure 5:**
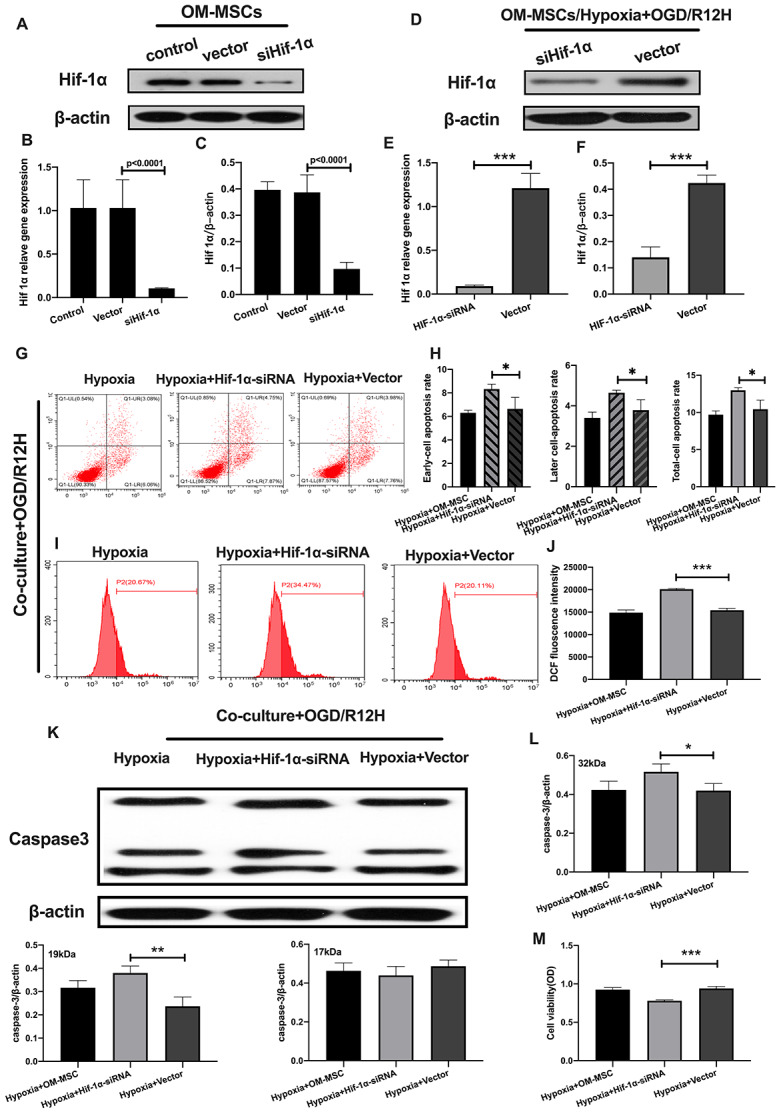
**Knockdown of HIF-1α in OM-MSCs aggravated cerebral OGD/R-induced apoptosis in BV2 microglial cells under hypoxic conditions.** (**A**–**C**) Successful knockdown of HIF-1α in OM-MSCs was verified by western-blot and qPCR. (**D**–**F**) The expression of HIF-1α in OM-MSCs was evaluated by western-blot and qPCR. (**G**, **H**) The apoptosis rate of BV2 microglial cells was evaluated by flow cytometry with Annexin V/PI staining. (**I**, **J**) Production of ROS in BV2 microglial cells was evaluated by flow cytometry. (**K**, **L**) The expression of caspase3 in BV2 microglial cells was quantified by Western blotting analysis. (**M**) The viability of BV2 microglial cells was assessed by MTT assay. All data are presented as the mean value ±SD. *p<0.05; **p<0.01, ***p<0.001, compared with vector group.

Having found that hypoxia preconditioned OM-MSCs attenuated apoptotic and pyroptotic cell death in BV2 microglial cells following OGD/R injury. We further investigated the role of HIF-1α in OM-MSCs under hypoxia conditions and its effect on different subtypes of microglial cell deaths. Knocking down of HIF-1α in OM-MSCs resulted in a significant reduction of protein and gene expression of HIF-1α-siRNA-transfected OM-MSCs group relative to the group transfected with the empty vector and controls, even under hypoxia-precondition (Figure 5A–5F). We found the rate of cell apoptosis and ROS were higher in BV2 microglial cells exposed to OGD/R for 12 hours and co-cultured with HIF-1α-siRNA-transfected OM-MSCs under hypoxia than in cells transfected with the empty vector OGD/R12-h after insult (Figure 5G–5J). Further, knock-down of HIF-1α increased the expression of caspase-3 and decreased the viability of BV2 microglial cells in the OM-MSCs co-culture group (Figure 5K–5M).

**Figure 6:**
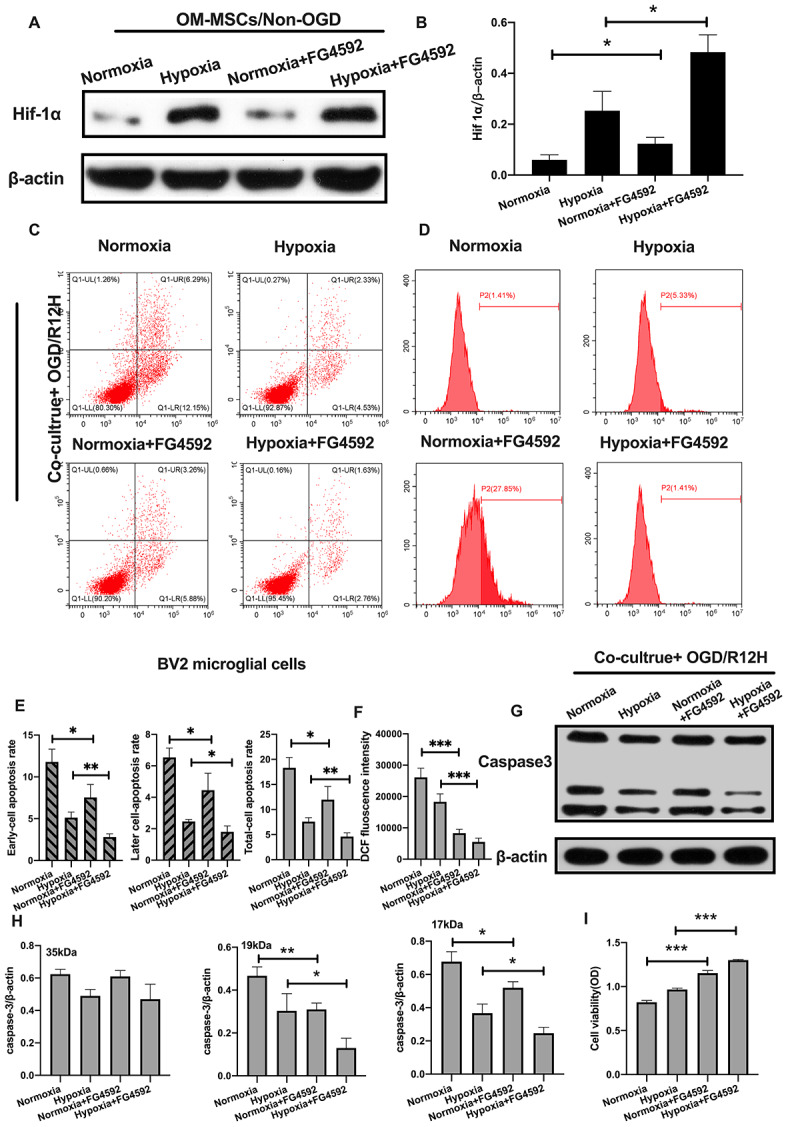
**Induction of HIF-1α in OM-MSCs by FG-4592 inhibited cerebral OGD/R-induced apoptosis in BV2 microglial cells.** (**A**, **B**) The successful overexpression of HIF-1α in OM-MSCs was confirmed by Western blotting. (**C**, **E**) The apoptosis rate of BV2 microglial cells was determined by flow cytometry with Annexin V/PI staining in each group. (**D**, **F**) Production of ROS levels in BV2 microglial cells was measured by flow cytometry. (**G**, **H**) The protein expression of caspase3 in BV2 microglial cells was quantified by Western blotting analysis. (**I**) The viability of BV2 microglial cells was evaluated with MTT assay. All data are presented as the mean value ±SD. *p<0.05; **p<0.01, ***p<0.001, compared with normoxia group or hypoxia group.

To further explore the role of HIF-1α in apoptosis of BV2 microglial cells exposed to OGD/R injury, we induced the expression of HIF-1α using FG-4592 in OM-MSCs, and then co-cultured these cells with BV2 microglial cells exposed to normoxia or hypoxia conditions [27]. HIF-1α levels were highest in BV2 microglial cells pretreated with the hypoxia-preconditioned OM-MSCs compared to normoxia group, normoxia incubated with FG4592 group and hypoxia preconditioned group (Figure 6A, 6B). Next, we explored whether FG-4592 treatment could prevent apoptosis in BV2 microglial cells co-cultured with OM-MSCs in the context of OGD/R12-h insult. Under normoxia conditions, the rate of cell apoptosis and ROS production were significantly lower in BV2 microglial cells after induction of HIF-1α relative to cells treated with control vector in the context of OGD/R insult for 12 hours. Furthermore, FG-4592 pretreatment promoted the suppression effects to maximum in hypoxia OM-MSCs co-culture group compared to the pretreatment with a non-inducer (Figure 6C–6F). Meanwhile, OM-MSCs pretreated with FG-4592 decreased expression of caspase3 in BV2 microglial cells under normoxia or hypoxia conditions in the context of OGD/R for 12 hours (Figure 6G, 6H). In addition, the cell viability of BV2 microglial cells co-cultured with OM-MSCs combined with FG-4592 exposed to hypoxia or normoxia conditions was higher compared to untreated cells under OGD/R injury for 12 hours (Figure 6I). Collectively, these results indicated that upregulation of HIF-1α expression in OM-MSCs attenuated apoptotic cell death suggesting a possible critical role in hypoxia-preconditioned OM-MSCs under OGD/R induced apoptosis in BV2 microglial cells.

### HIF-1α plays a crucial role in pyroptosis of BV2 microglial cells under OGD/R condition

**Figure 7:**
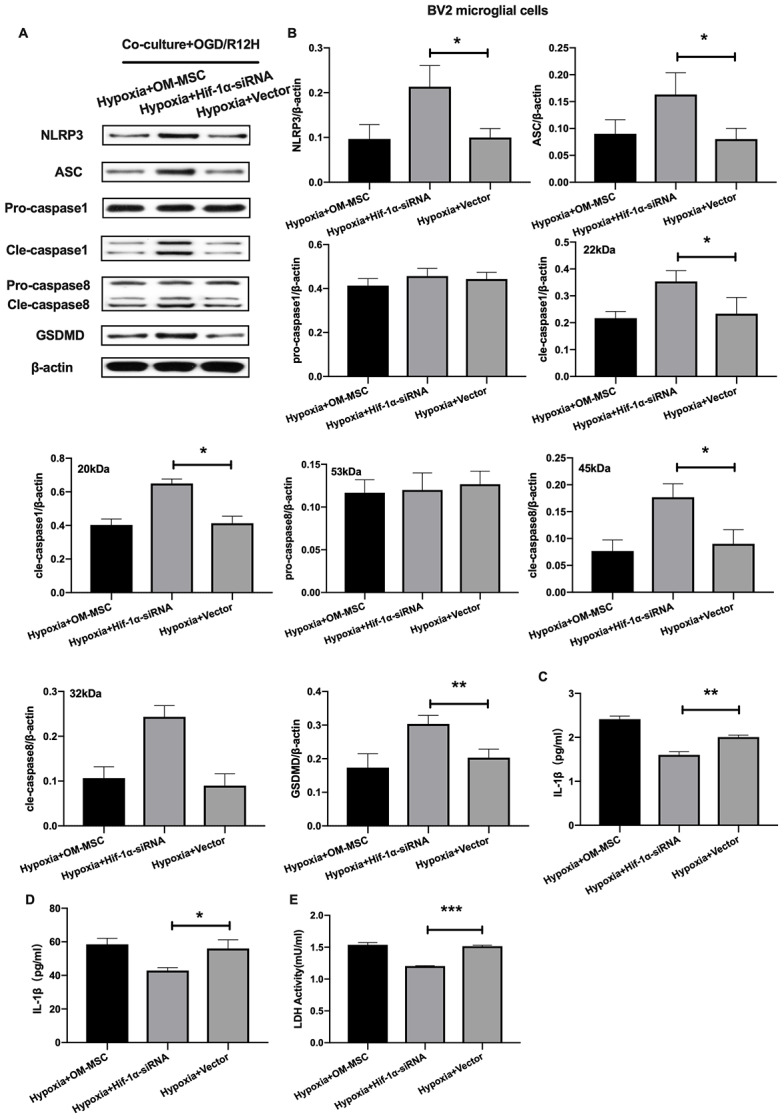
**Knockdown of HIF-1α in OM-MSCs exacerbated cerebral OGD/R-induced pyroptosis in BV2 microglial cells under hypoxic conditions.** (**A**, **B**) Expression of NLRP3, ASC, pro-caspase1, cleaved-caspase1, pro-caspase8, cleaved-caspase8 and GSDMD in BV2 microglial cells were quantified by Western blotting analysis. (**C**, **D**) Level of IL-1β and IL-18 in BV2 microglial cells were measured by ELISA. (**E**) The levels of LDH activity in BV2 microglial cells was determined by ELISA. All data are presented as the mean value ±SD. *p<0.05; **p<0.01, ***p<0.001, compared with vector group.

Given that HIF-1α plays an important role in apoptosis, we further investigated whether this factor also affects pyroptosis following OGD/R-induced injury in BV2 microglial cells subjected to OGD/R-induced injury. Results showed that NLRP3 inflammasome and pyroptosis-associated proteins levels were significantly higher after HIF-1α knocked-down. In contrast, pro-caspase1 and pro-caspase8 were not significantly upregulated after HIF-1α knocked-down (Figure 7A, 7B). Co-culture of BV2 microglial cells with siRNA-HIF-1α-transfection OM-MSCs increased the expression level of IL-1β and IL-18 relative to cells co-cultured with OM-MSCs transfected with an empty vector (Figure 7C, 7D). In addition, HIF-1α knock-down in OM-MSCs elevated LDH activity (Figure 7E).

**Figure 8:**
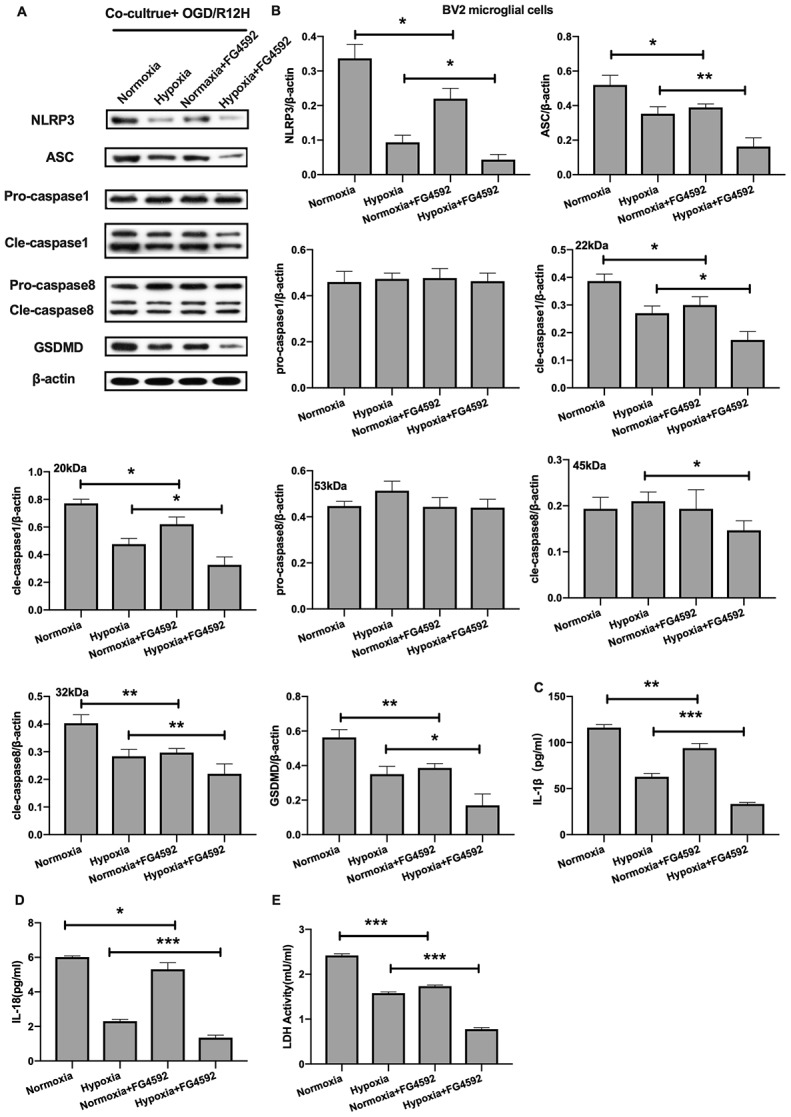
**Induction of HIF-1α in OM-MSCs by FG-4592 suppressed cerebral OGD/R-induced pyroptosis in BV2 microglial cells.** (**A**, **B**) Expression of NLRP3, ASC, pro-caspase1, cleaved-caspase1, pro-caspase8, cleaved-caspase8 and GSDMD in BV2 microglial cells was evaluated by Western blotting assay. (**C**, **D**) Level of IL-1β and IL-18 in BV2 microglial cells was quantified by ELISA. (**E**) The level of LDH activity in BV2 microglial cells was measured by ELISA. All data are presented as the mean value ±SD. *p<0.05; **p<0.01, ***p<0.001, compared with normoxia group or hypoxia group.

On the other hand, induction expression of HIF-1α with FG-4592 inhibited the expression of NLRP3 inflammasome and pyroptosis-associated proteins in BV2 microglial cells co-cultured with OM-MSCs preconditioned with normoxia condition relative to controls, in the context of OGD/R insult for 12 hours (Figure 8A, 8B). Furthermore, except of pro-caspase1 and pro-caspase8, the reduction in expression of NLRP3 inflammasome and pyroptosis-associated proteins observed in BV2 microglial cells co-cultured with hypoxia preconditioned OM-MSCs was significantly exacerbated upon exposure to hypoxia. The levels of IL-1β and IL-18 were higher in the normoxia-preconditioned OM-MSCs group, but decreased upon FG-4592 treatment in cells exposed to OGD/R injury for 12 hours. Co-culture of BV2 microglial cells with OM-MSCs treated with FG-4592 plus hypoxia markedly accelerated the reduction of IL-1β and IL-18 levels in BV2 microglial cells exposed to OGD/R for 12 hours, accompany with the reduction of LDH activity (Figure 8C–8E). Therefore, these findings suggested that HIF-1α may also play an important role in pyroptosis in BV2 microglial cells under OGD/R conditions. Taken together, these findings revealed that hypoxia-preconditioned OM-MSCs attenuated apoptosis or pyroptosis in BV2 microglial cells under cerebral ischemia/reperfusion conditions by regulating the expression levels of HIF-1α in *vitro*.

## DISCUSSION

Cerebral ischemia/reperfusion injury is a complicated pathological condition with unclear pathomechanisms. Studies have indicated that inflammation contributes to the development of cerebral ischemia/reperfusion injury [3]. Pyroptosis, a novel mode of inflammation-associated cell death, has been implicated in several diseases of the CNS [28]. Pyroptosis-induced programmed cell death is characterized by DNA fragmentation, in a similar fashion to apoptotic cell death [4]. Pyroptosis is also distinct from apoptosis, in that it is not regulated by the classical apoptotic-associated, and caspase-3 proteins, but by inflammation-associated protein caspase-1 [29]. Therefore, pyroptosis is also defined as caspase-1-dependent cell death. Pyroptosis comprises two pathways; canonical and non-canonical pathways. The canonical pathway is activated in non-infectious inflammation-associated diseases [30]. Several studies indicate that cerebral ischemia/reperfusion is involved in the canonical non-infectious pathway of pyroptosis in multiple cell types, including vascular endothelial cells, neurons, and astrocytes involves the canonical non-infectious pathway of pyroptosis [31–33]. However, to date, it is not known whether co-culturing OM-MSCs with microglia has a protective effect against either pyroptotic or apoptotic cell death during cerebral I/R injury.

Microglia play an important role in neuroinflammation as they release of pro- and anti-inflammatory mediators. In fact, previous studies have demonstrated that activation of microglial regulates the NLRP3 inflammasome is involved in activation of microglial [6]. During development of pyroptosis, NLRP3 is formed, and together with other important compounds of the inflammasome complex, acts to promote recruitment of inflammasome adaptor protein, ASC [34]. Induction of the inflammasome complex activates caspase-1 and facilitates formation of GSDMD, which further promotes secretion of pro-inflammation cytokines IL-1β and IL-18 from the pore of the cell membrane resulting in inflammation-associated pyroptotic cell death [5, 35]. It has been reported that activation of inflammasome induces apoptosis [36]. Apoptosis is a highly mediated subtype of cell death, which is particularly linked to the cerebral reperfusion phase. It differs from pyroptosis in that it results in cellular shrinkage and caspase-3 dependent cell death [29]. Pyroptosis induces inflammatory response, whereas apoptosis results from inflammatory response [4]. In this study, we investigated whether pyroptosis and apoptosis are involved in cerebral I/R conditions in microglia cells in *vitro*.

We first explored whether OGD/R injury induced apoptotic cell death in microglia cells. Results indicated that the rate of cell apoptosis increased and cell viability decreased during reperfusion. Moreover, OGD/R injury upregulated caspase3 expression and ROS production inBV2 microglia cells. These findings, therefore, provided evidence that apoptotic cell death was induced under cerebral OGD/R conditions in microglia cells.

We further examined NLRP3 inflammasome activation in BV2 microglia cells exposed to OGD/R injury. Notably, expression of ASC, pro-caspase1, cleaved-caspase1 and cleaved-caspse8 proteins were increased after OGD/R-induced insult. One of the typical characteristics of pyroptosis is the formation of a pore in the plasma membrane caused by GSDMD [10]. In our tests, GSDMD protein expression was significantly upregulated in BV2 microglia cells exposed to OGD/R-induced insult. In addition, IL-1β and 1L-18 levels increased after injury. LDH leakage from cellular membranes was enhanced upon OGD/R injury consistent with previous reports on ischemic injury [9]. It was also noted that inflammatory response occurred along the course of cerebral ischemia and reperfusion. In addition, pyroptotic cell death of microglia cells occurred during the ischemic or reperfusion stages. Taken together, our results demonstrate that OGD/R insult can induce pyroptotic and apoptotic cell death in BV2 microglia cells. Thus, inhibition of both processes in microglia upon cerebral ischemia/reperfusion injury may be an effective treatment approach for cerebral ischemic stroke.

Numerous studies have demonstrated the neuroprotective and immuno-regulatory effects of MSCs on diseases affecting the CNS [16]. For instance, Tang et al. [37] reported that MSCs can maintain the blood-brain barrier integrity by regulating the expression of aquaporin-4 protein, following cerebral ischemic stroke. However, it is not known whether MSCs can inhibit the newly described inflammatory form of programmed cell death, pyroptosis, in cerebral ischemia/reperfusion-induced insult remains unknown. Since OM-MSCs are unique source of MSCs, they could be an ideal treatment for ischemia/reperfusion related injuries [19]. But several optimization steps are needed to expedite the clinical utility of MSCs-based therapies. Specifically, the low survival and engraftment rates of transplanted MSCs and limited sources should be addressed [16]. Previous studies have reported that hypoxic preconditioning before engrafting MSCs effectively prevented apoptosis [23, 38]. Previously, we showed that hypoxic preconditioning of OM-MSCs increased secretion of paracrine factors which provided neuroprotection against cerebral I/R injury [21]. In this study, we found that co-culture with hypoxia preconditioned OM-MSCs significantly reduced apoptotic and pyroptotic cell death in BV2 microglial compared to normal OM-MSCs following cerebral OGD/R injury. Also, hypoxia preconditioned OM-MSCs decreased the secretion of pro-inflammation mediators (IL-1β and IL-18). This suggests that hypoxic preconditioning may be an effective approach to improve the anti-pyroptosis and anti-apoptosis effects of OM-MSCs.

Based on the aforementioned findings, we further investigated the mechanisms by which hypoxia preconditioning of OM-MSCs affected different subtypes of cell death. Several studies have reported that HIF-1α is a key nuclear transcription factor regulating responses to hypoxic conditions [26]. For instance, HIF-1α expression influences stemness, differentiation, and proliferation of MSCs [39]. However, it remains to be seen whether HIF-1α can affect pyroptosis and apoptosis in BV2 microglial cells under cerebral I/R condition. In this study, hypoxia preconditioned OM-MSCs markedly increased HIF-1α expression. Furthermore, silencing HIF-1α in OM-MSCs significantly upregulated caspase3 expression and accelerated rate of apoptosis. Moreover, silencing of HIF-1α resulted reduced cell viability and increased the expression of NLRP3 inflammasome and pyroptosis-associated proteins as well as LDH activity, IL-1β and IL-18 levels in response to OGD/R injury. These results indicate that HIF-1α can influence pyroptosis and apoptosis of BV2 microglial cells upon OGD/R insult. We further induced the expression of HIF-1α in OM-MSCs via FG-4592 pretreatment and preconditioned to hypoxia and normoxia. The results showed that induction of HIF-1α attenuated pyroptosis and apoptosis in BV2 microglial cells upon OGD/R challenge. Collectively, these results suggest that hypoxic preconditioning and induction of HIF-1α expression in OM-MSCs can protect against pyroptosis and apoptosis induced by cerebral I/R injury.

In conclusion, this study demonstrates that OGD/R induced pyroptosis and apoptosis in BV2 microglial cells. Hypoxia preconditioning of OM-MSCs enhances HIF-1α expression, herby providing neuroprotection against pyroptotic and apoptotic cell death in microglial cells. Thus, OM-MSCs can be used to treat cerebral ischemic stroke.

## MATERIALS AND METHODS

### Ethics statement and primary culture of human OM-MSCs and BV2 microglial cell

Human OM-MSCs were isolated from the interior surface of concha nasalis media from healthy donors (three males and one female), aged 18-50 years old. This was done by otolaryngology endoscopy operation at the Department of Otolaryngologic Surgery, the second affiliated hospital of Hunan Normal University (Changsha, China). Informed consent was obtained from each subject before operation. All protocol were approved by the ethics committee of Hunan Normal University.

OM-MSCs were isolated and cultured as described previously [40]. Briefly, the obtained human OM tissues were washed 5 times with penicillin–streptomycin (Invitrogen, Carlsbad, CA, USA) under 37°C. The tissues were then cut into approximately 0.5mm^3^ blocks and cultured in Dulbecco's modified Eagle's medium comprising nutrient mixture F12 (DMEM/F12; Invitrogen) and10% fetal bovine serum (FBS; Invitrogen, USA) at 37°C in a 5% CO_2_ atmosphere. OM-MSCs at the fifth passage were used for experiments. Hypoxia preconditioning was performed as previously described [21, 41]. Briefly, OM-MSCs were cultured in a chamber (Billups Rothenberg, Inc., Del Mar, CA) containing 1% oxygen, 5% CO_2_ and balanced N_2_ at 37°C atmosphere for 48 hours. BV2 microglial cells were purchased from the Cell Storage Center of the Chinese Academy of Sciences (Shanghai, China) and cultured in Dulbecco's modified Eagle's medium (DMEM, Invitrogen, USA) containing 10% fetal bovine serum (FBS; Invitrogen, USA) supplemented with 100 U/ml penicillin and 100 μg/ml streptomycin and maintained at 37°C under a 5% CO_2_ atmosphere.

### Identification of OM-MSCs

OM-MSCs were identified by flow cytometry based on presence of the following cell surface molecules; Nestin, STRO-1, CD34, CD44, CD45, CD73, CD90, CD105, CD133, and CD146. All antibodies used in this experiment are outlined in Supplementary Table 1.

### Oxygen-glucose deprivation and reperfusion (OGD/R) model

The Culture medium was replaced with D-hank's balanced salt solution (Biological Industries, USA) prior to induction of OGD/R injury in BV2 microglial cells. The OGD/R injury model was created by culturing the cells in a special chamber (Billups Rothenberg, Inc., Del Mar, CA) containing 5% CO_2_, and 95% N_2_, set at 37°C for 6 hours. Thereafter, the cultured cells were removed from the chamber and replaced with fresh culture medium and continuously cultured at 37°C under 5% CO_2_ atmosphere in different time points to mimic reperfusion processes.

### Co-culture of OM-MSCs with BV2 microglial cells

Transwell co-culture system plates (Corning, USA) were used to co-culture BV2 microglial cells with OM-MSCs for 24 hours. The co-cultured cells were used for subsequent experiments according to previously described protocols [42].

### LDH activity assay

To examine the integrity of cell membranes and release of cellular contents, we measured a LDH activity assay using the LDH activity assay kit (Beyotime, China) according to the manufacturer's instructions.

### Determination of cell viability and apoptosis

Viability of BV-2 microglia cells was detected using the MTT assay kit (Sigma-Aldrich, St. Louis, MO, USA). Absorbance of the platewas recorded at 450nm using a microplate reader (Themo-fisher, USA). Apoptosis of BV2 microglia cells was measured using FITC Annexin V apoptosis detection kit (KeyGen Biotech, Jiangsu, China) as previously described and detected by flow cytometry (FACSCalibur, Becton-Dickinson, Sunnyvale, CA).

### Intracellular ROS assay

Intracellular ROS levels were measured using ROS kit (Beyotime, China) and flow cytometry (FACSCalibur, Becton-Dickinson, Sunnyvale, CA) according to the manufacturer's instructions.

### Determination of pro-inflammation cytokines levels

Levels of pro-inflammation cytokines **,** IL-1β and IL-18 were detected using ELISA kits (Beyotime, China) according to manufacturer's instructions. Respective absorbances were recorded at 450nm using a microplate reader (Themo-fisher, USA).

### Western blot analysis

Western blotting was performed as previously described [42]. Briefly, the cell samples were lysed using RIPA Lysis Buffer (Beyotime, China) according to the manufacturer's instruction. The concentration of the proteins was determined using the BCA protein assay kit (Beyotime, China). All primary and secondary antibodies used in this experiment are outlined in Supplementary Table 2.

### Knockdown of HIF-1α by small interfering RNA (siRNA)

The siRNA knockdown of HIF-1α expression, the following siRNA target sequences of HIF-1αwere used: CCCATTCCTCATCCGTCAAAT. These are primer sequence were selected for mRNA quantification: forward; TCCAGCAGACCCAGTTACAGA, and reverse; GCCACTGTATGCTGATGCCTT. The forward sequence of Actin was: ACATCCGTAAAGACCTCTATGCC, and the reverse sequence of Actin was: TACTCCTGCTTGCTGATCCAC. The expression of HIF-1α in OM-MSCs was silenced using siRNA transfection kit according to the manufacturer's instructions (Ribobio, China). The efficiency of HIF-1α knock-down in OM-MSCs was verified by Western blotting and qPCR assays.

### Induction of HIF-1α expression

To induce the expression of HIF-1α in OM-MSCs, the inducer FG-4592 (APEBIO, USA) was transfected into cells as described in the manufacturer's protocol. The success of induction of HIF-1α was determined by measuring the highest mRNA expression of HIF-1α.

### Statistical analysis

All experimental data are presented as the mean ± standard deviation (SD). Comparisons among groups were performed by t-test or analysis of variance (ANOVA). *P* value < 0.05 was considered statistically significant. All analyses were performed using Prism GraphPad 8.0.

## Supplementary Material

Supplementary Figure 1

Supplementary Tables
