# Correction for: Hypoxia-preconditioned olfactory mucosa mesenchymal stem cells abolish cerebral ischemia/reperfusion-induced pyroptosis and apoptotic death of microglial cells by activating HIF-1α

**DOI:** 10.18632/aging.204321

**Published:** 2022-10-14

**Authors:** Yan Huang, Fengbo Tan, Yi Zhuo, Jianyang Liu, Jialin He, Da Duan, Ming Lu, Zhiping Hu

**Affiliations:** 1 Key Laboratory of Protein Chemistry and Developmental Biology of Ministry of Education, College of Life Sciences, Hunan Normal University, Changsh, Hunan, 410081, P.R. China; 2 Department of Neurosurgery, Second Affiliated Hospital of Hunan Normal University, Changsh, Hunan, 410003, P.R. China; 3 Hunan Provincial Key Laboratory of Neurorestoration, Second Affiliated Hospital of Hunan Normal University, Changsh, Hunan, 410003, P.R. China; 4 Department of Gastrointestinal Surgery, Xiangya Hospital, Central South University, Changsh, Hunan, 410008, P.R. China; 5 Department of Neurology, The Second Xiangya Hospital, Central South University, Changsh, Hunan, 410011, P.R. China

**Keywords:** hypoxia-preconditioned OM-MSCs, HIF-1α, microglial, pyroptosis, apoptosis

**This article has been corrected:** The authors requested replacement of **Figure 6,** in which the images in **panel D** – production of ROS in BV2 microglial cells in the Normoxia group (the upper right subpanel) and Hypoxia+FG4592 group (the bottom left subpanel) – were incorrectly placed during assembly of the figures. This resulted in duplication of the image of ROS production in the Hypoxia+FG4592 group (P2=1.41%). The authors corrected **Figure 6D** by using the correct flow cytometry data/image from the original sets of experiments for the Normoxia group (P2=34.68%). This correction does not affect the article's conclusions. The authors would like to apologize for any inconvenience caused.

New **Figures 6** is presented below.

**Figure 6:**
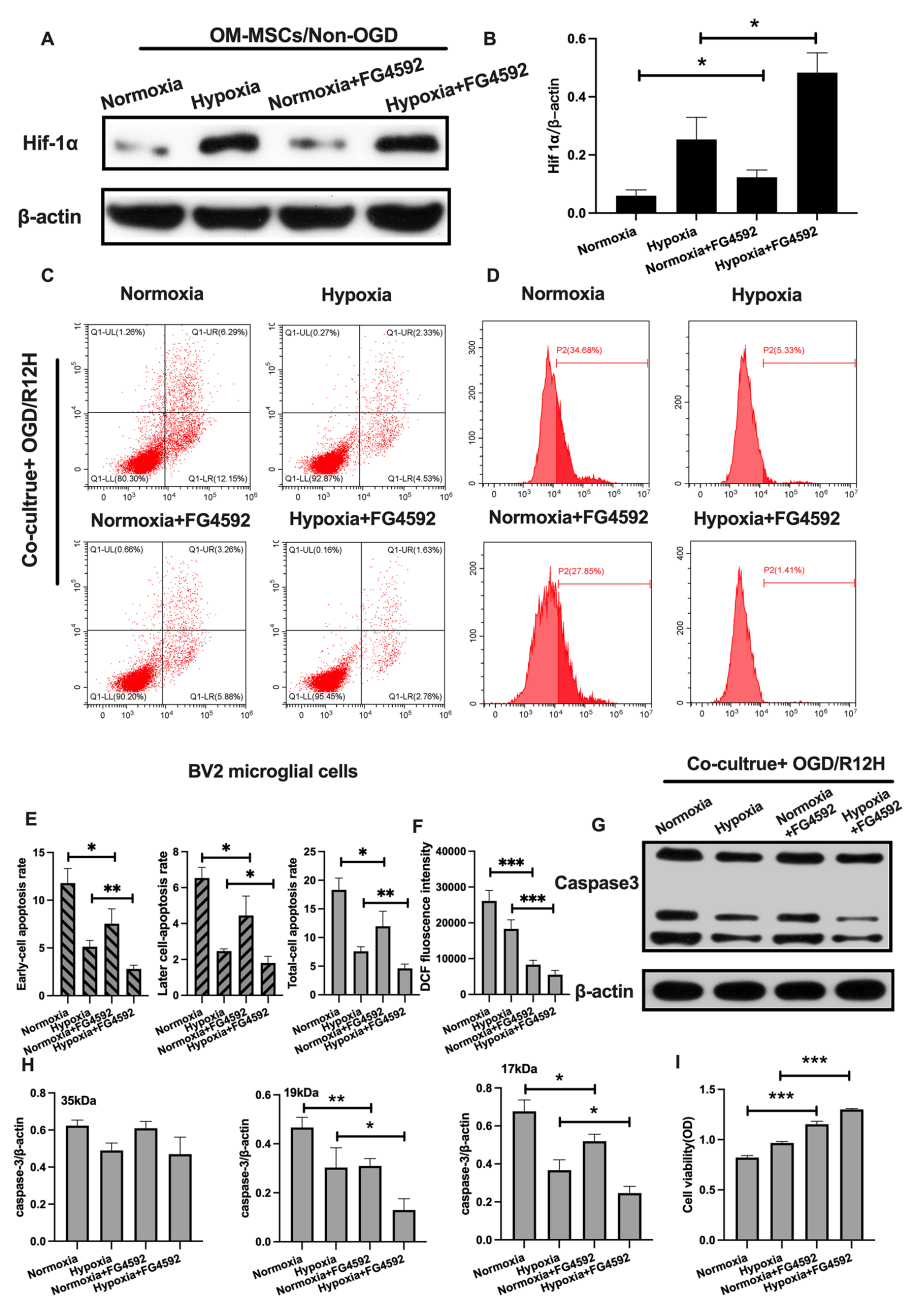
**Induction of HIF-1α in OM-MSCs with FG-4592 inhibited cerebral OGD/R-induced apoptosis in BV2 microglial cells.** (**A**, **B**) The successful overexpression of HIF-1α in OM-MSCs was confirmed by Western blotting. (**C**, **E**) The apoptosis rate among BV2 microglial cells was determined with flow cytometry and Annexin V/PI staining in each group. (**D**, **F**) Production of ROS levels in BV2 microglial cells was measured with flow cytometry. (**G**, **H**) Protein expression of caspase3 in BV2 microglial cells was quantified by Western blotting. (**I**) The viability of BV2 microglial cells was evaluated with MTT assays. All data are presented as the mean ±SD. *p<0.05; **p<0.01, ***p<0.001, compared to the normoxia or hypoxia group.

