# Supplementary material for: Hypoxia-preconditioned olfactory mucosa mesenchymal stem cells abolish cerebral ischemia/reperfusion-induced pyroptosis and apoptotic death of microglial cells by activating HIF-1α
Source: Aging (Albany NY). 2020 Jun 7;12(11):10931–50. doi: 10.18632/aging.103307 (PMC7346036; doi:10.18632/aging.103307)
Supplement: Supplementary Figure 1 [file aging-12-103307-s001..pdf]

SUPPLEMENTARY FIGURE

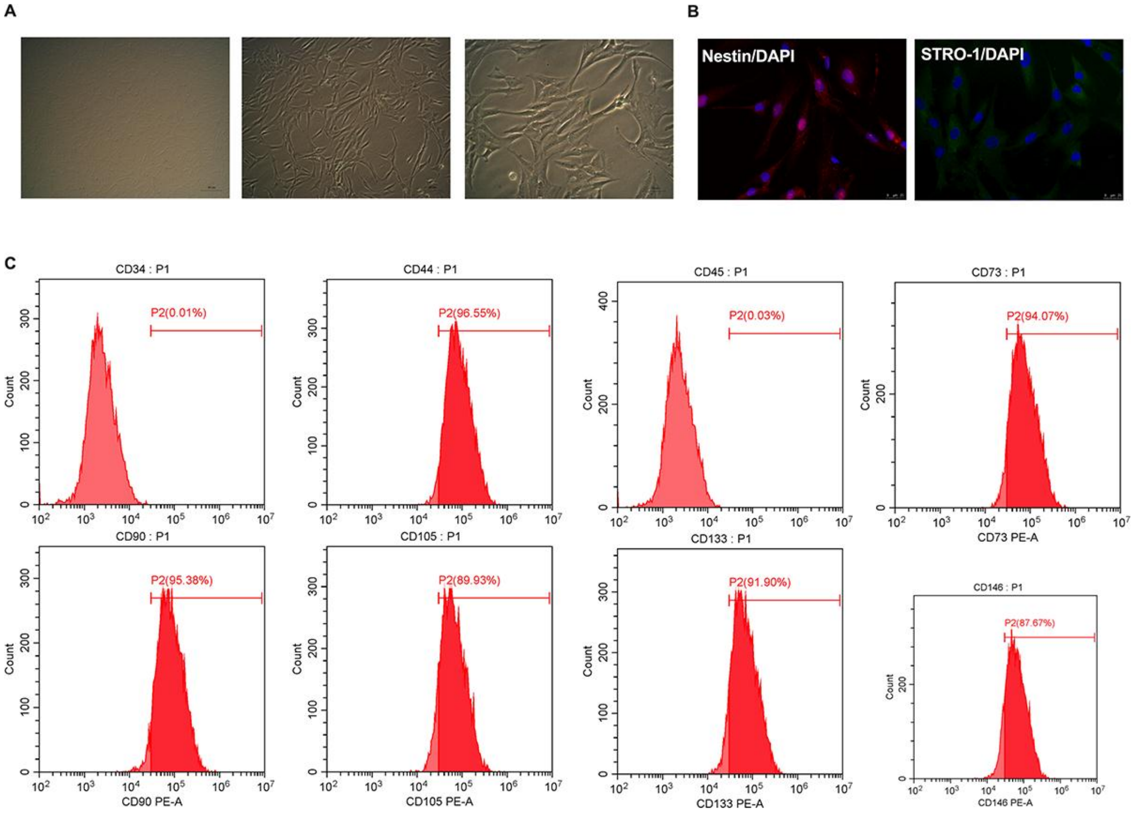

Supplementary Figure 1. Characterization of OM-MSCs. (**A**) The morphology of OM-MSCs obtained from human olfactory mucosa (OM) (magnification, ×40, ×100, ×200). (**B**) Immunofluorescent labeling were positive for Nestin and STRO-1(scale bar,25μm). (**C**) The flow cytometry assesses for the immunophenotypic marker of OM-MSCs in the fifth passage.
