# Supplementary material for: Hypoxia-preconditioned olfactory mucosa mesenchymal stem cells abolish cerebral ischemia/reperfusion-induced pyroptosis and apoptotic death of microglial cells by activating HIF-1α
Source: Aging (Albany NY). 2020 Jun 7;12(11):10931–50. doi: 10.18632/aging.103307 (PMC7346036; doi:10.18632/aging.103307)
Supplement: Supplementary Tables [file aging-12-103307-s002..pdf]

SUPPLEMENTARY TABLES

Supplementary Table 1. The list of antibodies used for immunofluorescence and flow cytometry.

Product	Catalogue Number	Supplier
immunofluorescence:		
Nestin	DF7754	Affinity Biosciences, Golden, CO, USA
STRO-1	14-6688-82	Invitrogen, Carlsbas, CA
flow cytometry:		
CD34	130-113-741	Miltenyi Biotec,Germany
CD45	130-110-770	Miltenyi Biotec,Germany
CD44	130-113-904	Miltenyi Biotec,Germany
CD73	130-112-060	Miltenyi Biotec,Germany
CD90	130-114-902	Miltenyi Biotec,Germany
CD105	130-098-906	Miltenyi Biotec,Germany
CD133	130-113-670	Miltenyi Biotec,Germany
CD146	130-097-939	Miltenyi Biotec,Germany

Supplementary Table 2. Primary and secondary antibodies.

Product	Catalogue Number	Supplier
Primary antibody:		
WB:		
anti-caspase-3	19677-1-AP	Proteintech, Chicago, IL, USA
anti-GSDMD	ab219800	Abcam, Cambridge, MA, USA
anti-ASC	bs-6741R	Bioss, Beijing, China
anti-NLRP3	ab214185	Abcam, Cambridge, MA, USA
anti-Caspase1(Pro and Cleaved) ab207802	Abcam, Cambridge, MA, USA	
anti-Caspase8(Pro and Cleaved) 19677-1-AP	Proteintech, Chicago, IL, USA	
anti-HIF-1α	20960-1-AP	Proteintech, Chicago, IL, USA
β-actin	60008-1-Ig	Proteintech, Chicago, IL, USA
Secondary antibody:		
WB:		
anti-mouse IgG	SA00001-1	Proteintech, Chicago, IL, USA
anti-rabbit IgG	SA00001-2	Proteintech, Chicago, IL, USA
